# Biofeedback Intervention for Stress and Anxiety among Nursing Students: A Randomized Controlled Trial

**DOI:** 10.5402/2012/827972

**Published:** 2012-06-28

**Authors:** Paul Ratanasiripong, Nop Ratanasiripong, Duangrat Kathalae

**Affiliations:** ^1^Department of Advanced Studies in Education and Counseling, California State University, Long Beach, CA 90840, USA; ^2^Student Health Services, California State University, Long Beach, CA 90840, USA; ^3^Department of Nursing, Boromarajonani College of Nursing, Nakhornratchasima 30000, Thailand

## Abstract

*Purpose.* It has been well documented that nursing students across the world experience stress and anxiety throughout their education and training. The purpose of this randomized controlled study is to investigate the impact of biofeedback intervention program on nursing students' levels of stress and anxiety during their first clinical training. *Methods.* Participants consisted of 60 second-year baccalaureate nursing students. The 30 participants in the biofeedback group received training on how to use the biofeedback device to assist in stress and anxiety management for 5 weeks while the 30 in the control group did not receive any training. *Findings.* Results indicated that the biofeedback group was able to maintain the stress level while the control group had a significant increase in the stress level over the 5-week period of clinical training. Additionally, the biofeedback group had a significant reduction in anxiety, while the control group had a moderate increase in anxiety. *Conclusions.* The better the nursing students can manage their stress and anxiety, the more successful they can be in their clinical training. Ultimately, the more psychologically healthy the nursing students are, the more likely they will flourish and graduate to become productive and contributing members of the nursing profession.

## 1. Introduction

It has been well documented that nursing students across the world experience stress and anxiety throughout their education and training [1–7]. Issues that contribute to their stress and anxiety include academic challenges, clinical challenges, technological advances, financial concerns, interpersonal difficulties, family problems, physical and mental health issues, inadequate support, and poor coping skills. Additional stressors for nonnative nursing students include cultural adjustments, language issues, social isolation, and discrimination.

 Furthermore, during the initial clinical training experience, nursing students report increases in their levels of stress and anxiety [8–13]. The stress and anxiety levels increase as nursing students learn to apply their theoretical knowledge to the clinical work with their first patients in new environments while being observed by their clinical instructors and their peers.

 Over the past 2 decades, many forms of interventions have been suggested to help nursing students with their stress and anxiety. One study developed a 6-week individualized stress-management program to help reduce performance anxiety of nursing students [14]. Another study utilized a worksite 6-session relaxation intervention workshop to help reduce the anxiety level of nursing students [15]. Furthering the implementation of relaxation practices, a study found that mindfulness meditation over 8-week timeframe is helpful in reducing stress and anxiety among nursing students [4]. Most recently, one researcher recommended using humor, peer instructors, mentors, and mindfulness training to help decrease nursing students' anxiety [11]. Another research found cognitive modification with biofeedback techniques for self-relaxation helpful in reducing anxiety in nursing students over a 5-week period [16].

 However, no recent studies have been conducted with the new generation of portable biofeedback equipments to help address nursing students' stress and anxiety specifically during the beginning of the clinical training period. The purpose of this randomized controlled research study is to investigate the impact of an early biofeedback intervention program on the levels of stress and anxiety of second-year baccalaureate nursing students as they begin their first clinical experience.

## 2. Background

### 2.1. Stress and Anxiety

Stress can be described as “physical, mental, or emotional strain or tension” as well as “a condition or feeling experienced when a person perceives that demands exceed the personal and social resources the individual is able to mobilize” [17]. Excessive stress interferes with the person's ability to function normally. According to Spielberger, states of anxiety are characterized by subjective feelings of nervousness, worry, tension, or apprehension, and by arousal of the autonomic nervous system (i.e., sweating, heart palpitation, muscle tension) [18]. Excessive anxiety also interferes with the person's ability to function normally.

 Both stress and anxiety are ubiquitous among nursing students. Mimura et al. [19] found that nursing students in Japan and England have additional vulnerability to stress as well as higher level of stress when compared with the general college-student population. Tully [7] reported significant distress among nursing students in Ireland and found that second-year nursing students were significantly more distressed than first-year students.

 During the first clinical experience of nursing students, stress and anxiety have been reported to be very high. Yucha et al. [20] reported that anxiety levels among nursing students in clinical setting were high compared to those nonnursing college students in the United States. Another study found high anxiety during clinical practice among nursing students in Canada [21]. Jimenez et al. [22] found that nursing students in Spain experience clinical training stressors more intensely than academic or external stressors and display more psychological symptoms than physiological symptoms. Specific stressors for nursing students as they encounter the new clinical rotation include fear of the unknown, implementation of technical skills, fear of specific rotation, and the writing of care plans [23].

### 2.2. Biofeedback

Biofeedback is a process of becoming aware of the body's physiological functions. Using specialized devices and sensors, the person can receive feedback on heart rate, skin temperature, brainwave activity, blood pressure, respiration, and muscle activity. Biofeedback training helps the person learn to modify physiological activity to improve health and performance [24]. Biofeedback training has been utilized to help with various conditions including anxiety, asthma, attention deficit hyperactivity disorder, chronic pain, depression, epilepsy, headache, hypertension, insomnia, irritable bowel syndrome, posttraumatic stress disorder, stroke, and urinary incontinence [25]. Different modalities of biofeedback training are used for the different conditions. Some of the widely used modalities include electromyographic (EMG), heart rate variability (HRV), electrocardiogram (ECG), electroencephalograph (EEG), electrodermograph (EDA), and feedback thermometer.

 The early use of biofeedback training with nursing students was in the 1970s. One study utilized EMG feedback training with nursing students in Ireland to help reduce anxiety [26]. Since then, there have been only a few studies that used biofeedback training with nursing students. Ohkuma examined the effects of evoking imagery to control skin temperature with biofeedback among nursing students in Japan [27]. Utz used auditory biofeedback training to lower muscle tension in nursing students in the United States while Prato helped American nursing students reduce test anxiety with a biofeedback-assisted relaxation training program [28, 29]. Thus far, no study has focused specifically on using biofeedback training with nursing students to manage stress and anxiety as they begin their clinical training.

 For the present study with nursing students in clinical settings, portability and ease of use are the two important factors in selecting the biofeedback training type and device. Based on the previous review article of portable biofeedback devices [30], this study selected the emWave PSR device that uses the heart rate variability (HRV) measurement. HRV is the beat-to-beat variation in the heart rate. Measuring the individual's HRV is a noninvasive process as doing so only requires placement of the thumb on the pulse sensor of the portable emWave PSR device. In 3 steps, HRV biofeedback training helps the individual to (1) become aware of the involuntary HRV, (2) learn to control the HRV through slower breathing and positive emotions, and (3) achieve a heart-rhythm pattern associated with lower stress and anxiety-related symptoms.

## 3. Methods

### 3.1. Design and Participants

This randomized controlled study was conducted with second-year baccalaureate nursing students at a public nursing college in Thailand. The purpose of this research was to investigate the impact of biofeedback intervention on the stress and anxiety levels of nursing students as they begin their clinical training.

 The participants for this study consisted of 60 second-year baccalaureate nursing students. All of the participants were female. The age range for participants was between 18 and 21 years old (*M* = 19.27, *SD* = 0.61). The GPA range for participants was between 2.98 and 3.90 (*M* = 3.46, *SD* = 0.22).

 Sixty participants were selected based on a priori power analysis by G*Power computer program [31]. Using parameters of 0.8 power, 0.75 moderate effect size, and 0.05 alpha, the sample size for*t*-test needed per group was 29 participants.

### 3.2. Procedures and Instruments

Once the nursing college's institutional review board approval was obtained, nursing students were recruited to participate in the study. Participants for this study were all adult volunteer nursing students. After the volunteer participants signed the informed consent forms, they were randomly assigned to either the biofeedback intervention group or the control group. All participants completed the pre-intervention and post-intervention surveys consisting of brief demographic information, Perceived Stress Scale, and State Anxiety Scale. The preintervention survey was completed at the beginning of the new school year, before classes and clinical training began. The post-intervention survey was conducted 5 weeks after the pre-intervention survey. An incentive of 150 Baht (local currency equivalent to 5 moderate meals) was given to each participant who volunteered for the study.

 After completing the preintervention survey, the 30 participants in the biofeedback group received 2 training sessions led by the researchers on how to use the portable biofeedback device to assist in stress and anxiety management. Specifically, participants were trained to control the HRV through slower breathing and positive emotions. The portable biofeedback device provided immediate visual and auditory feedback to the participants to help them learn to control the HRV. After completing the training and being able to achieve a sustained heart-rhythm pattern associated with lower stress and anxiety-related symptoms, each nursing student in the biofeedback group was given a portable biofeedback device to use for 5 weeks. They were instructed to use the portable device for biofeedback training 3 times per day and record their practice time on the log. The 30 participants in the control group did not receive any training or device to use.

 The Perceived Stress Scale (PSS) was used to assess participants' level of perceived stress in the past month [32]. The PSS contains 10 items using a 5-point Likert scale (0 = never, 4 = very often). A higher score indicates a higher level of perceived stress. Several previous studies have used the PSS with nursing students [19, 33, 34]. The internal consistency (Cronbach's alpha) of the Perceived Stress Scale for the current sample was  .77 for the preintervention and  .80 for the postintervention.

 The State Anxiety Scale from the State-Trait Anxiety Inventory was used to assess participants' current level of anxiety [18]. The State Anxiety Scale contains 20 items using a 4-point Likert scale (0 = not at all, 3 = very much so). A higher score indicates a higher level of current anxiety. Several previous studies have use the State Anxiety Scale with nursing students [6, 15, 35]. The internal consistency (Cronbach's alpha) of the State Anxiety Scale for the current sample was  .93 for the preintervention and  .91 for the postintervention.

## 4. Results

No significant differences in age and GPA were found between the biofeedback group and the control group (see Table 1). There were no significant differences in the preintervention Perceived Stress Scale and the preintervention State Anxiety Scale between the two groups.

### 4.1. Stress

**Figure 1:**
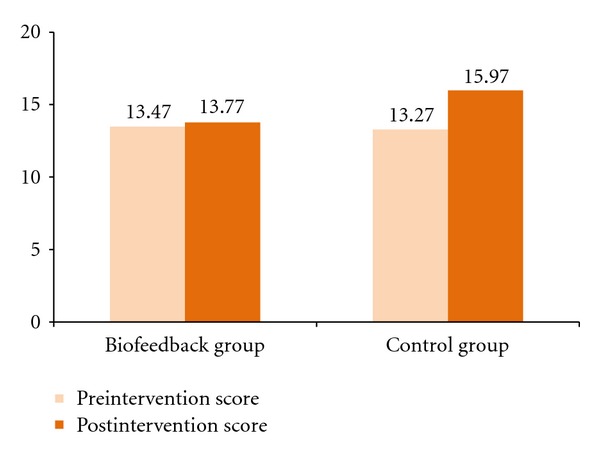
Pre- and postintervention mean scores for Perceived Stress Scale.

In terms of stress, the biofeedback group had a very small and nonsignificant increase in the Perceived Stress Scale score over the 5-week period, while the control group had a significant increase (see Figure 1). For the biofeedback group, the mean postintervention PSS score (*M* = 13.77, *SD* = 4.64) only slightly increased from the mean preintervention PSS score (*M* = 13.47, *SD* = 4.26). For the control group, the mean postintervention PSS score (*M* = 15.97, *SD* = 4.37) significantly increased from the mean preintervention PSS score (*M* = 13.27, *SD* = 4.32). A paired-sample *t*-test for the control group indicated a significant increase in the PSS score: *t*(29) = 3.74, *p* < .001. The Cohen's *d* = 0.62, a medium effect.

### 4.2. Anxiety

**Figure 2:**
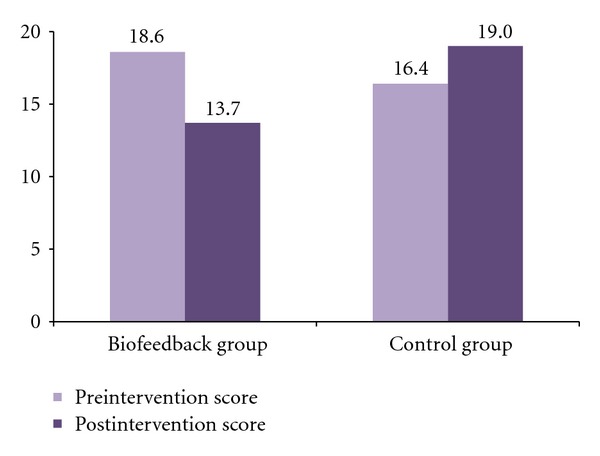
Pre- and postintervention mean scores for State Anxiety Scale.

In the area of anxiety, the biofeedback group had a significant decrease in the State Anxiety Scale score over the 5-week period while the control group had a moderate increase (see Figure 2). For the biofeedback group, the mean postintervention state anxiety score (*M* = 13.70, *SD* = 6.70) was significantly lower than the mean preintervention state anxiety score (*M* = 18.60, *SD* = 10.25). A paired-sample *t*-test for the biofeedback group indicated a significant decrease in the State Anxiety Scale score: *t*(29) = 2.93, *p* < .01, Cohen's *d* = 0.57 (a medium effect). For the control group, the moderate increase from the mean preintervention state anxiety score (*M* = 16.40, *SD* = 8.34) to the mean postintervention state anxiety score (*M* = 19.00, *SD* = 8.69) was not statistically significant. When comparing the two groups, the biofeedback group had a significantly lower level of anxiety than the control group after the 5-week intervention, *t*(58) = 2.65, *p* < .01, Cohen's *d* = 0.68, a medium effect.

## 5. Discussion

Based on the findings from previous studies, both stress and anxiety levels are expected to increase for nursing students when they begin their first clinical training if they do not receive any interventions. The results from this study demonstrate that the 5-week biofeedback training intervention not only kept the nursing students' stress levels from increasing, but also significantly reduced their levels of anxiety.

 Nursing students in the Biofeedback Group were able to maintain the same level of stress over the 5-week period even though they experienced more stressors and demands from their new clinical training. Nursing students in the Control Group had a significant increase in their stress level over the same period. Additionally, nursing students in the Biofeedback Group had a significant decrease in their anxiety level over this 5-week period while those in the control group had a moderate increase in anxiety level.

### 5.1. Implications

It is very important for nurse educators to help nursing students manage their stress and anxiety in order to prevent additional problems. Ross et al. [36] found that nursing students who experience high stress tend to be depressed. High stress and anxiety in nursing students also negatively affect learning and academic performance by impeding memory, concentration, and problem solving skills [37]. Depression in nursing students often affects their ability to perform their clinical duties, their relationship with the patients, and their attitude toward the nursing profession. Likewise, if nursing students experience problems with concentration and problem solving, not only will they have a hard time getting through school, but also will they not be performing their nursing duties at optimum levels, which will affect the quality of patient care.

 The positive results of the biofeedback training program from this study give nurse educators another tool to help augment the clinical training component of nursing education. With limited investment in time and financial resources, a biofeedback training program could be set up to help improve the psychological well-being of nursing students as they begin their clinical training. Furthermore, nursing students will gain awareness of how the body works and how the body and mind are linked, ultimately providing them with a more comprehensive, participatory understanding of their patients.

 In addition to using biofeedback training to help reduce anxiety, Suliman and Halabi [6] also found that critical thinking and self-esteem were negatively related to state anxiety. Nurse educators can train students in critical thinking skills as well as set up programs to help improve students' self-esteem to reduce the anxiety levels among nursing students. With an increased sense of self-efficacy via biofeedback training or critical thinking skills along with the mastery of each subject or clinical area through education and training, nursing students can keep stress and anxiety at bay and perform according to their potential.

### 5.2. Limitations and Future Research

This study was conducted only at one nursing college in one country. Future studies should consider replicating the study at multiple nursing colleges and multiple countries. Additionally, a follow-up study on the impact of the biofeedback training on stress, anxiety, and academic performance of nursing students after one to two years will help nurse educators to better understand the long-term efficacy of the biofeedback intervention program.

## 6. Conclusion

With clinical training being one of the most vital components of the nursing education, it is imperative that nurse educators continue the effort to help nursing students manage their stress and anxiety during this important process. Biofeedback training is one tool that has been demonstrated to be effective in helping with this developmental journey. The better the nursing students can manage their stress and anxiety, the more successful they can be in their clinical training. Ultimately, the more psychologically healthy the nursing students are, the more likely they will flourish and graduate to become productive and contributing members of the nursing profession.

## Figures and Tables

**Table 1:** Demographics and pretest scores for biofeedback group and control group.

Variable	Biofeedback group (*n* = 30)		Control group (*n* = 30)		*P*
	*M*	*SD*	*M*	*SD*	
Age	19.30	0.70	19.23	0.50	*ns*
GPA	3.46	0.20	3.45	0.25	*ns*
Stress	13.47	4.26	13.27	4.32	*ns*
Anxiety	18.60	10.25	16.40	8.34	*ns*

*ns*: not significant.
